# A Novel Multimodal Approach to Point-of-Care Ultrasound Education in Low-Resource Settings

**DOI:** 10.5811/westjem.2020.4.45928

**Published:** 2020-07-09

**Authors:** Andrea Dreyfuss, David A. Martin, Angel Farro, Robert Inga, Sayuri Enríquez, Daniel Mantuani, Arun Nagdev

**Affiliations:** * Alameda Health System, Department of Emergency Medicine, Oakland, California; † Hospital Nacional Dos de Mayo, Department of Emergency Medicine, Cercado de Lima, Peru

## Abstract

Point-of-care ultrasound (POCUS) enables physicians to make critical diagnosis and treatment decisions at the bedside. However, access to and expertise with this technology remain limited in Peru. Establishing longitudinal POCUS educational curriculums in remote, low-resource settings can be challenging due to geographical distances, encumbering the ability to provide ongoing hands-on support. Previously described educational interventions have focused on training individual users on clinical applications of POCUS, rather than training physicians how to teach POCUS, thereby limiting scalability and sustainable impact. We therefore describe our experiences establishing the first ultrasound fellowship curriculum in Peru, which incorporates tele-ultrasonography to circumvent traditional geographical barriers.

## INTRODUCTION

Point-of-care ultrasound (POCUS) is recognized as an integral skill set for emergency medicine (EM) providers working in the United States (US).^1^ The World Health Organization considers ultrasound as one of the most important technologies for developing countries; however, a lack of education and training remains a limiting factor to widespread use.^2^

Prior studies have demonstrated the ability to teach clinical applications of emergency care POCUS to novices with adequate retention of knowledge and skills when longitudinal educational curriculums are employed.^3^–^5^ However, sustainability and scalability of longitudinal educational programs is resource intensive, and often not possible. International travel for instructors for hands-on education is expensive and time consuming. Also, prior studies have focused on training large groups of individual users, rather than educating future physician leaders on how to teach POCUS. Our novel model is an attempt to allow for scalability and ongoing impact.

Recent technological advances (both improved, broadband Internet access as well as ultrasound software advances) allow for tele-ultrasonography to be used as an additional tool for providing ongoing supervision and mentoring of learners.^6^ Prior studies have demonstrated that tele-ultrasonography can be employed to direct image acquisition by novice providers^7^ and also increase diagnostic accuracy when combined with expert mentorship.^8^

EM has been a recognized medical subspecialty in Peru since 1993. While most emergency departments (ED) have access to an ultrasound system due to a national decree from the Ministry of Health in 2015 requiring all EDs treating critically ill patients to have access to an ultrasound, many remain unused due to lack of training. Given this identified need for further EM POCUS training, we formed a partnership with the Department of EM at the Hospital Nacional Dos de Mayo to establish the first EM ultrasound fellowship in Peru. The Hospital Nacional Dos de Mayo is a large, urban, public academic medical center that primarily serves the uninsured. Prior to the initiation of our educational project, the ED had an ultrasound system devoted to clinical care that was primarily used for focused assessment with sonography for trauma (FAST) examinations.

In this report we describe our experiences creating the first ultrasound fellowship for emergency physicians in Peru. Our educational model leverages tele-ultrasonography to provide ongoing remote education and support, alongside traditional in-person hands-on education. Our model is aimed at providing fellows with sufficient depth of education and expertise to become educators and future leaders within the field of EM POCUS.

## METHODOLOGY

Three Peruvian, EM-trained doctors were selected as the inaugural class of ultrasound fellows based on recommendations by faculty and residents at the Hospital Nacional Dos de Mayo. Participation in the fellowship was voluntary. The year-long fellowship is modeled after the ultrasound fellowship curriculum at our academic institution and incorporates traditional in-person, hands-on learning both in Lima and the US with real-time tele-ultrasonography, educational teleconferences, and academic journal reviews. The educational intervention was reviewed by the Alameda Health System Institutional Review Board and deemed to be exempt from any informed consent requirements.

### Hands-on Education

The fellowship begins with a two-week interactive bootcamp course held in Lima covering basic and advanced ultrasound topics both with lectures and hands-on scanning component on models and patients. The course is taught by visiting ultrasound faculty from our academic institution. After the introductory course, fellows begin to perform clinically relevant ultrasounds in the ED at the Hospital Nacional Dos de Mayo. Fellows participate in three, four-hour-long weekly ultrasound scanning shifts during which they are protected from primary patient care responsibilities. During the initial two months, ultrasound scanning shifts were led by visiting ultrasound instructors from our academic institution. Following this initial period, visiting instructors are not always present in Lima; however, fellows use the built-in tele-ultrasonography software to obtain guidance with image acquisition and interpretation.

Additionally, fellows travel separately as early as possible in their fellowship year to our US-based medical center to spend a one month-long rotation with our EM ultrasound division. They participate in daily ultrasound scanning shifts led by ultrasound faculty and learn advanced POCUS diagnostic and procedural skills. During the rotation, fellows also observe how POCUS education is incorporated into medical school and residency education, which is critical to our model’s sustainability and scalability through the teaching of future educators.

### Tele-ultrasonography

**Figure 1:**
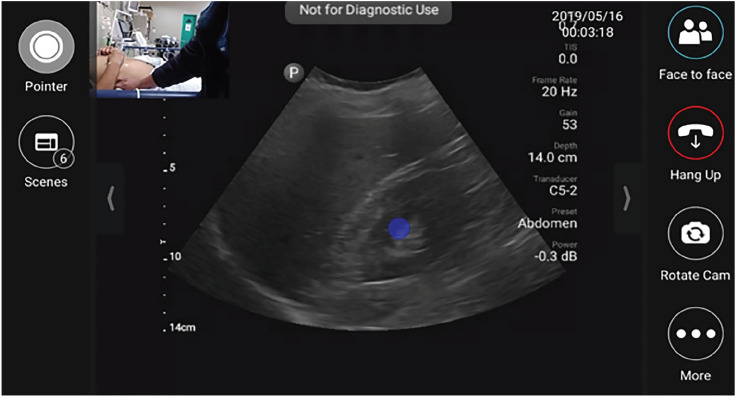
Tele-ultrasound platform as visualized by remote user providing diagnostic/procedural assistance. The blue dot is an indicator that can be manipulated by either user.

Fellows employ tele-ultrasonography to provide real-time, remote supervision and guidance using built-in software on the handheld ultrasound devices during scanning shifts. The tele-ultrasound software allows the fellows to join real-time video chat with a pool of on-call ultrasound faculty and alumni from our academic medical center in the US while performing ultrasound scans. Fellows are encouraged to call when there is diagnostic uncertainty or need for additional procedural support. Ultrasound faculty and alumni can connect to the tele-ultrasound application on their personal cell phone, tablet, or computer and have access to real-time footage of both the ultrasound images, as well as a video camera that can be used to visualize and direct placement of the ultrasound probe and/or guide procedures as shown in Figure 1.

### Educational Teleconferences

Educational teleconferences occur weekly in Spanish to review ultrasound images for quality assurance and to discuss scientific articles related to POCUS. Fellows upload de-identified ultrasound images and video clips onto an online storage database and videos are reviewed during the educational teleconferences. Fellows provide their initial ultrasound interpretations by annotating the media on the online data-storage platform, and the images are subsequently reviewed for both image quality and image accuracy of initial interpretation by faculty from our EM ultrasound division. Ultrasound faculty can also provide asynchronous feedback by annotating the uploaded media prior to the weekly educational teleconference.

After reviewing all of the Peruvian fellows’ images, the fellows join in the division of ultrasound’s weekly quality assurance conference at our academic institution. The discussion is in English; however, simultaneous translation is provided via the chat feature of the teleconferencing platform by one of our two native Spanish-speaking, ultrasound faculty members.

## RESULTS

**Figure 2:**
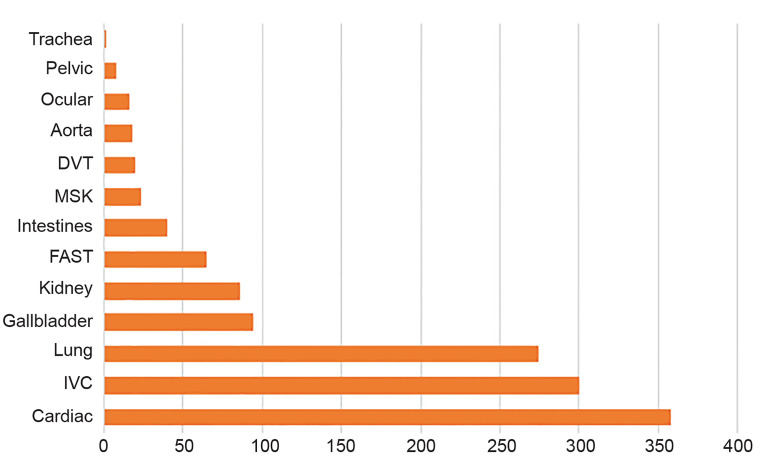
Graphical representation of total ultrasound studies performed by category by three fellows during the fellowship year. *DVT*, Deep venou thrombosis; *MSK*, musuloskeletal; *FAST*, focused assessment of sonography in trauma; *IVC*, inferior vena cava.

**Figure 3:**
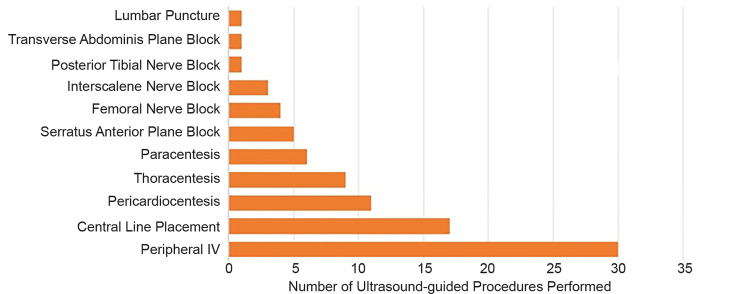
Graphical representation of ultrasound-guided procedures performed by inaugural class of three emergency medicine ultrasound fellows in Lima, Peru.

Our fellows uploaded over 1300 ultrasound studies to our online database during the year-long fellowship representing over 500 hours of clinically protected time dedicated to performing ultrasounds. The studies encompass multiple images and have been categorized into either diagnostic or procedural scans. Figure 2 provides a graphical representation of the total number of ultrasound studies performed by category for the entire fellowship year. Cardiac, inferior vena cava, and lung ultrasound represent over 70% of all studies performed. Fellows have additionally performed over 80 ultrasound-guided procedures as evidenced in Figure 3. The most common ultrasound procedural application has been vascular access.

Each fellow has completed a one-month ultrasound rotation at our academic institution including more than 80 hours of ultrasound scanning time with experienced ED sonographers. Fellows have participated on average in over 190 hours of quality assurance and educational teleconferences with our local US division.

Fellows have presented at both national and international conferences on advanced POCUS applications such as regional pain anesthesia and management of cardiac arrest. Since completion of the fellowship, one of our graduates has published an ultrasound case report in a US-based EM journal on the POCUS features of late-stage Ebstein’s anomaly findings. Additionally, the fellows created the first annual ultrasound procedural course for EM residents in Peru. Over 40 EM residents participated in the conference held at the Hospital Nacional Dos de Mayo in December 2018. They have also developed an elective ultrasound rotation for EM residents nationwide and have had a total of 12 rotators since the creation of the elective, with plans to begin to accept international rotators from throughout Latin America. In addition to developing a resident curriculum, the fellows are in the process of developing ongoing, one-on-one training sessions with local faculty to ensure a basic understanding of POCUS within the entire EM faculty.

The fellowship program has also led to interdepartmental collaborations with multiple specialties including general surgery, trauma, orthopedics, and cardiology. For example, the general surgeons have requested that our fellows perform POCUS on patients presenting with abdominal pain, since they previously primarily relied on history and exam findings due to limited access to computed tomography. The trauma and orthopedic services have also embraced the use of ultrasound-guided regional nerve anesthesia for the treatment of acute pain in patients with traumatic injuries.

## DISCUSSION

In this report we describe the unique aspects of our educational model for teaching POCUS to emergency physicians in Peru. While previous studies have primarily focused on teaching general practitioners POCUS applications using traditional hands-on education,^3^–^5^ we employ an innovative multimodal approach to train future POCUS educators and leaders. Our approach uses a novel multi-pronged approach using tele-ultrasonography to provide ongoing remote education and support, in addition to traditional hands-on education.

Previously described limitations with global health ultrasound education projects include geographical distances and language barriers.^5^ We have been able to limit the burden of international travel by recruiting multiple visiting instructors and employing tele-ultrasonography to provide ongoing remote education. Additionally, fellows spend one month of the year at our home institution in the US, which allows them to gain firsthand clinical experience in a setting where POCUS is already integrated within the clinical workflow of the ED. We have mitigated potential language barriers by developing a curriculum that is executed in Spanish. All of our visiting instructors are fluent in Spanish and teleconferencing sessions are held in Spanish with the exception of our division of ultrasound quality assurance conference, which has simultaneous translation provided.

We have been able to reduce financial costs associated with running our program by using ultrasound machines equipped with tele-ultrasonography software on loan for the initial year of the fellowship from Philips Healthcare. Travel costs for fellows have been partially reimbursed by using scholarship funds from our local EM resident international group. While fellows do not receive an additional stipend for participating in the fellowship, they were able to reduce their overall clinical burden and obtain protected ultrasound scanning time due to our partnership with hospital administration at the Hospital Nacional Dos de Mayo.

## LIMITATIONS

The ability to replicate this project is contingent on political buy-in from key stakeholders abroad. Part of our success has been due to the time and effort spent building relationships with the local hospital and the Peruvian national EM society. Thus, the generalizability of our project to other low-resource settings is contingent on building similar partnerships.

## CONCLUSION

Our program is unique compared to other international POCUS educational interventions because fellows become POCUS experts and leaders in ultrasound education at the completion of their fellowship year. We expect fellows to become educational leaders for effecting curricular change and integration of POCUS within graduate medical education in Peru. To that extent, the graduates from the inaugural class are now our co-directors of the ultrasound fellowship program, helping us oversee the education of our second class of ultrasound fellows. We currently have four new ultrasound fellows and have expanded to three additional public academic hospital training sites. Two of the new sites are in smaller cities in more remote areas of Peru: Cusco in the Andean highlands and Iquitos in the Amazonian rainforest. We are now using tele-ultrasonography within Peru, employed by our inaugural class of graduates to provide remote support to our two new fellows outside of Lima. The scalability and sustainability of our program has been facilitated by training local champions who continue to grow the POCUS community within Peru and by leveraging tele-ultrasonography to reduce geographical barriers to ongoing education.
